# Overall Survival Prediction in Renal Cell Carcinoma Patients Using Computed Tomography Radiomic and Clinical Information

**DOI:** 10.1007/s10278-021-00500-y

**Published:** 2021-08-11

**Authors:** Zahra Khodabakhshi, Mehdi Amini, Shayan Mostafaei, Atlas Haddadi Avval, Mostafa Nazari, Mehrdad Oveisi, Isaac Shiri, Habib Zaidi

**Affiliations:** 1 Rajaie Cardiovascular Medical and Research Center, Iran University of Medical Science, Tehran, Iran; 2 Division of Nuclear Medicine and Molecular Imaging, Geneva University Hospital, CH-1211 Geneva 4, Switzerland; 3 Department of Biostatistics, School of Health, Kermanshah University of Medical Sciences, Kermanshah, Iran; 4 Epidemiology and Biostatistics Unit, Rheumatology Research Center, Tehran University of Medical Sciences, Tehran, Iran; 5 School of Medicine, Mashhad University of Medical Sciences, Mashhad, Iran; 6 Department of Biomedical Engineering and Medical Physics, Shahid Beheshti University of Medical Sciences, Tehran, Iran; 7 Department of Computer Science, University of British Columbia, Vancouver, BC Canada; 8 Comprehensive Cancer Centre, School of Cancer & Pharmaceutical Sciences, Faculty of Life Sciences & Medicine , Kings College London, London, UK; 9 Geneva University Neurocenter, Geneva University, Geneva, Switzerland; 10 Department of Nuclear Medicine and Molecular Imaging, University Medical Center Groningen, University of Groningen, Groningen, Netherlands; 11 Department of Nuclear Medicine, University of Southern Denmark, Odense, Denmark

**Keywords:** CT, Radiomics, Survival prediction, Machine learning, Renal cell carcinoma

## Abstract

The aim of this work is to investigate the applicability of radiomic features alone and in combination with clinical information for the prediction of renal cell carcinoma (RCC) patients’ overall survival after partial or radical nephrectomy. Clinical studies of 210 RCC patients from The Cancer Imaging Archive (TCIA) who underwent either partial or radical nephrectomy were included in this study. Regions of interest (ROIs) were manually defined on CT images. A total of 225 radiomic features were extracted and analyzed along with the 59 clinical features. An elastic net penalized Cox regression was used for feature selection. Accelerated failure time (AFT) with the shared frailty model was used to determine the effects of the selected features on the overall survival time. Eleven radiomic and twelve clinical features were selected based on their non-zero coefficients. Tumor grade, tumor malignancy, and pathology t-stage were the most significant predictors of overall survival (OS) among the clinical features (*p* < 0.002, < 0.02, and < 0.018, respectively). The most significant predictors of OS among the selected radiomic features were flatness, area density, and median (*p* < 0.02, < 0.02, and < 0.05, respectively). Along with important clinical features, such as tumor heterogeneity and tumor grade, imaging biomarkers such as tumor flatness, area density, and median are significantly correlated with OS of RCC patients.

**Supplementary Information:**

The online version contains supplementary material available at 10.1007/s10278-021-00500-y.

## Introduction

Renal cell carcinoma (RCC) accounts for 2 to 3% of all cancer types [1]. Worldwide, RCC is the tenth and sixth commonly diagnosed cancer in women and men, respectively [2]. In recent decades, RCC incidence has shown an increasing trend for both sexes [3], which can be attributed to recent advancements in imaging techniques, such as contrast-enhanced computed tomography (CECT) and magnetic resonance imaging (MRI). Besides, approximately more than 50% of RCC cases are diagnosed incidentally when abdominal imaging is performed for gastrointestinal disorders [4]. According to cancer statistics, the 5-year relative survival rate of RCC patients depends on the cancer stage at the time of diagnosis. The survival rate after 5 years is 93%, 69%, and 12% for localized tumors, tumors with regional lymph nodes metastasis, and tumors with distant metastasis, respectively [5]. From the clinical perspective, predictive and prognostic models have a pivotal role in treatment and management, precision medicine, and prediction of the overall cancer outcome [6]. Currently, the most important prognostic model and commonly accepted staging system for RCC is The American Joint Committee on Cancer (AJCC) tumor-node-metastasis (TNM) staging [7]. This system is limited to anatomic prognostic factors. Yet, the rapid expansion of our understanding of cancer biology and the development of novel effective treatments along with advancements in medical imaging technology have pushed researchers toward looking beyond the TNM staging system and developing new predictive models [7]. Recent studies have proposed prognostic factors, including histologic, clinical, genomic, and imaging features [8–11].

Radiomic and radiogenomic biomarkers have gained increasing popularity among researchers in recent years for developing diagnostic, prognostic, and predictive models [12–16]. Radiomic refers to the translation of medical images into minable high-dimensional data and identification of patterns from imaging data via data mining algorithms to improve clinical decision support systems [12–16]. A number of studies have demonstrated the potential of radiomic analysis in survival analysis and prediction of treatment outcome [12–16]. Oikonomou et al. [17] investigated the predictive power of radiomic features along with maximum standardized uptake value extracted from PET/CT images of lung cancer patients treated with stereotactic body radiotherapy (SBRT). They reported that radiomic features could have a complementary role in prognostication in lung cancer patients post-SBRT. Jiang et al. [18] reported that a radiomic signature containing 19 selected radiomic features captured from CT imaging is associated with disease-free survival (DFS) and overall survival (OS) in patients with gastric cancer. Park et al. [19] developed a radiomic nomogram for patients with invasive breast cancer. The developed nomogram consisted of radiomic signatures, MRI, and clinico-pathological factors. It improved DFS estimation in comparison with radiomic signatures or clinico-pathological nomogram alone.

In the context of kidney cancer, most of the conducted radiomic studies focused mainly on differentiating benign tumors, such as angiomyolipoma without visible fat and oncocytoma from malignant renal masses like RCC subtypes [20–22]. However, there are a limited number of studies that investigated the correlation between texture-based imaging features and RCC patients’ survival. Haider et al. [9] performed CT texture analysis on 40 RCC patients before and after treatment with Sunitinib to predict their progression-free survival (PFS) and OS. Their results showed that entropy from the CT texture (reflecting the heterogeneity in the texture) was a significant predictor of the patients’ overall survival for both before and after treatment. Moreover size-normalized standard deviation of the intensities in CT images (both pre-treatment and follow-up) was also associated with PFS and OS [9]. The aim of this work is to investigate the applicability of radiomic features and clinical data for the prediction of RCC patients’ overall survival after partial or radical nephrectomy.

## Materials and Methods

**Fig. 1:**
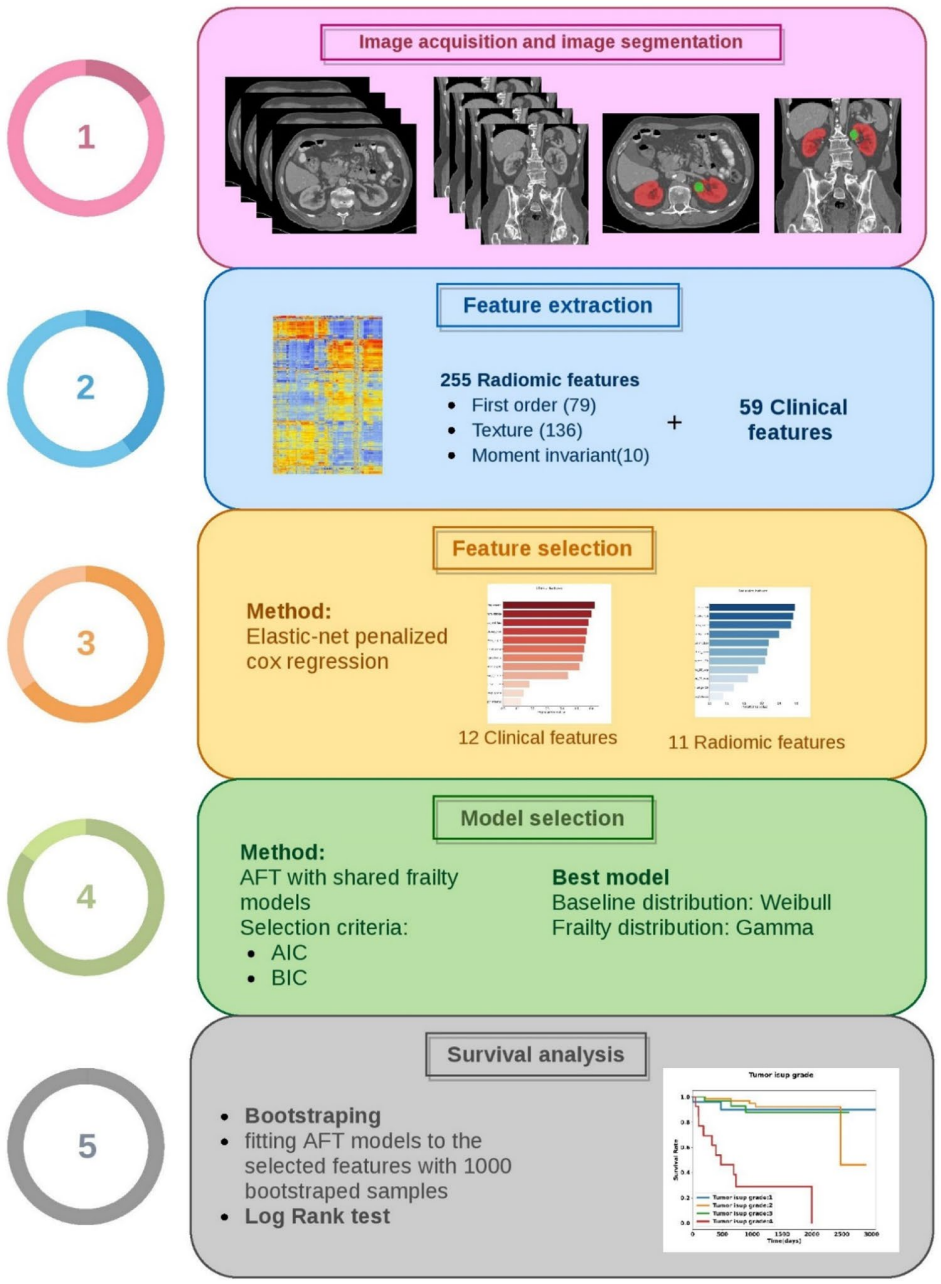
The radiomic workflow adopted in this study. AFT: accelerated failure time, AIC: Akaike information criteria, BIC: Bayesian information criteria

The workflow of the current study is illustrated in Fig. 1.

### Image Acquisition

The dataset used in this work was taken from The Cancer Imaging Archive (TCIA) and consists of CT scans in the arterial phase of 210 patients who underwent either partial or radical nephrectomy [23–25]. The summary of clinical data is provided in Supplemental Table 1 and more information could be found in previous studies [23–25]

### Image Segmentation

A region of interest (ROI) delineating each tumor was manually drawn under the supervision of a urological surgeon; more information could be found in previous studies [23–25].

### Feature Extraction

Images were interpolated to isotropic voxel spacing of 2 × 2 × 2 mm^3^ using cubic interpolation to obtain rotationally invariant texture features. Prior to feature extraction, intensity levels in images were discretized into 64 Gy-levels. Feature extraction was performed using The Standardized Environment for Radiomics Analysis (SERA) package [26]. This is a MATLAB-based framework for the calculation of standardized radiomic features compliant with the Imaging Biomarker Standardization Initiative (IBSI) [27, 28] used in multi-center standardization studies [27, 28]. In this work, a total of 225 radiomic features were extracted from each ROI including 79 first-order features (morphology, statistical, histogram, and intensity-histogram features), 136 three-dimensional textural features from texture matrices (GLCM, GLRLM, GLSZM, GLDZM, NGTDM, NGLDM), and 10 moment-invariant features. Supplemental Table 2 summarizes the details of the extracted features.

### Feature Selection

We used three sets of features, including radiomic, clinical, and radiomic + clinical features where all sets contained a large number of variables. Such high-dimensional data are predisposed to model overfitting, minimal-optimal problem, and increased computational time. Moreover, not all of the features are informative and even some may be irrelevant and do not contribute to the prediction of survival. Therefore, we used an elastic net penalized Cox regression to reduce the dimensionality of features and remove irrelevant features.

In medical research, one of the most commonly used models for investigating the association between covariates and survival data is the Cox proportional hazard model [29]. In a Cox model, the hazard function is calculated using the following equation:

1 $$h\left(t;{X}_{1},{X}_{2},\dots {X}_{p}\right)={h}_{0}\left(t\right)\mathrm{exp}\left({\beta }_{1}{X}_{1}+\dots +{\beta }_{p}{X}_{p}\right)$$

In this equation, ${h}_{0}(t)$ is a completely unspecified baseline hazard function, ${X}_{j}$ defines covariates (in our study radiomic and/or clinical features), and ${\beta }_{j}$ stands for associated coefficients for *j* = 1 … *p* (number of covariates or features). By maximizing the Cox partial likelihood function, the vector of coefficients $\beta$ can be approximated by

2 $$L\left(\beta \right)={\Pi }_{i=1\dots n S.t. {\delta }_{i}=1}\frac{\mathrm{exp}({\beta }^{T}{X}^{i})}{{\sum }_{l\in {R}_{i}}{\mathrm{exp}({\beta }^{T}{X}^{l})}^{^{\prime}}}$$

In the above equation, ${X}^{i}={({X}_{1}^{i},\dots ,{X}_{p}^{i})}^{T}$ is the vector of covariates for the ${i\mathrm{th}}$ patient, ${R}_{i}$ is the set of patients at risk at the time ${t}_{i}$, and if the event has been observed for the ${i\mathrm{th}}$ patient, then ${\delta }_{i}=1$. This regression model with many predictors may have high variance. To circumvent this problem, we can add a penalty term to log the partial likelihood of Cox model [30]. We used the elastic net penalty, which is a combination of ridge and LASSO penalties. By adding this penalty to the log partial likelihood, we get

3 $$\widehat{\beta }=\beta \mathrm{argmax}l\left(\beta \right)-\lambda (\alpha {\left|\left|\beta \right|\right|}_{1}+\frac{1-\alpha }{2}{\left|\left|\beta \right|\right|}_{2}^{2})$$

In the above equation, $l\left(\beta \right)=\mathrm{log}(L\left(\beta \right))$ is the log-partial-likelihood [31]. In this work, the optimal hyper-parameter ($\alpha )$ and the tuning parameter (*λ*) were determined using a ten-fold cross-validation. This process was implemented using the “glmnet” R package [30].

### Model Development and Survival Analysis

The two most important regression models in the context of survival analysis are Cox proportional hazard model [32] and accelerated failure time (AFT) model [33]. Unlike the Cox model which assumes that the effect of a covariate is to multiply the hazard by some constant, AFT models provide a linear relationship between the log of the failure time and the covariates and are favorable to studies in which some covariates may accelerate or decelerate the expected failure time [34]. The regression form of the AFT model is given by:

4 $$log{T}_{i}={{\beta }_{0}+\beta }_{1}{x}_{i1}+\dots {\beta }_{p}{x}_{ip}+\sigma {\varepsilon }_{i}$$

where ${T}_{i}$ denotes the failure time for the ${i\mathrm{th}}$ subject, ${x}_{1}\dots {x}_{p}$ are covariates with ${\beta }_{1}\dots {\beta }_{p}$ coefficients, $\sigma$ is the scale parameter, and ${\varepsilon }_{i}$ is a random variable. In the above equation, the failure time can have different distribution patterns, such as Weibull, log-normal, exponential, gamma, and log-logistic. Most of the survival analyses assume that the study population is homogeneous and all subjects are under the same risk. However, this assumption is often incorrect since the response to a specific treatment may be different among individuals [35, 36]. With respect to the aforementioned issue, the shared frailty survival model is one of the basic approaches that introduce random effects and unobserved heterogeneity into the model. Whenever the frailties become common between the clusters of subjects, shared frailty can be used to describe a random effect model [37]. By introducing the frailty to the AFT model, we are given by

5 $$log{T}_{ij}= {\omega }_{i}+{{\varvec{X}}}_{{\varvec{i}}{\varvec{j}}}^{\boldsymbol{^{\prime}}}{\varvec{\beta}}+\sigma {\varepsilon }_{ij}$$

Here, ${T}_{ij}$ denotes the lifetime of ${j\mathrm{th}}$ individual in the ${i\mathrm{th}}$ cluster or group, and $\mathrm{exp}{\omega }_{i}$ is the random frailty distributed within a cluster which can have different distributions [34].

To analyze the effects of the selected features on the overall survival time, we used accelerated failure time (AFT) with the shared frailty model. To identify the best model, we fitted AFT with shared frailty models with different baseline and frailty distributions to our data. The best model was selected according to the Akaike information criteria (AIC) [38] and the Bayesian information criteria (BIC) [39]. AIC and BIC are defined by the following equations:

6 $$\mathrm{AIC}=2k-2 \mathrm{log}(\widehat{L})$$

7 $$\mathrm{BIC}=k*\mathrm{log}\left(n\right)-2\mathrm{log}(\widehat{L})$$

In the above equations, *k* is the number of parameters estimated by the model, $\widehat{L}$ is the maximized value of the likelihood function of the model, and *n* is the number of observations. In the statistical analysis, a model with the lowest AIC and BIC is preferred. Following the identification of the optimum AFT shared-frailty model, we performed “survival” and “frailtypack” R packages with 1000 bootstrapping samples to reach a robust estimation of standard errors of regression coefficients [40, 41].

## Results

### Feature Selection

**Fig. 2:**
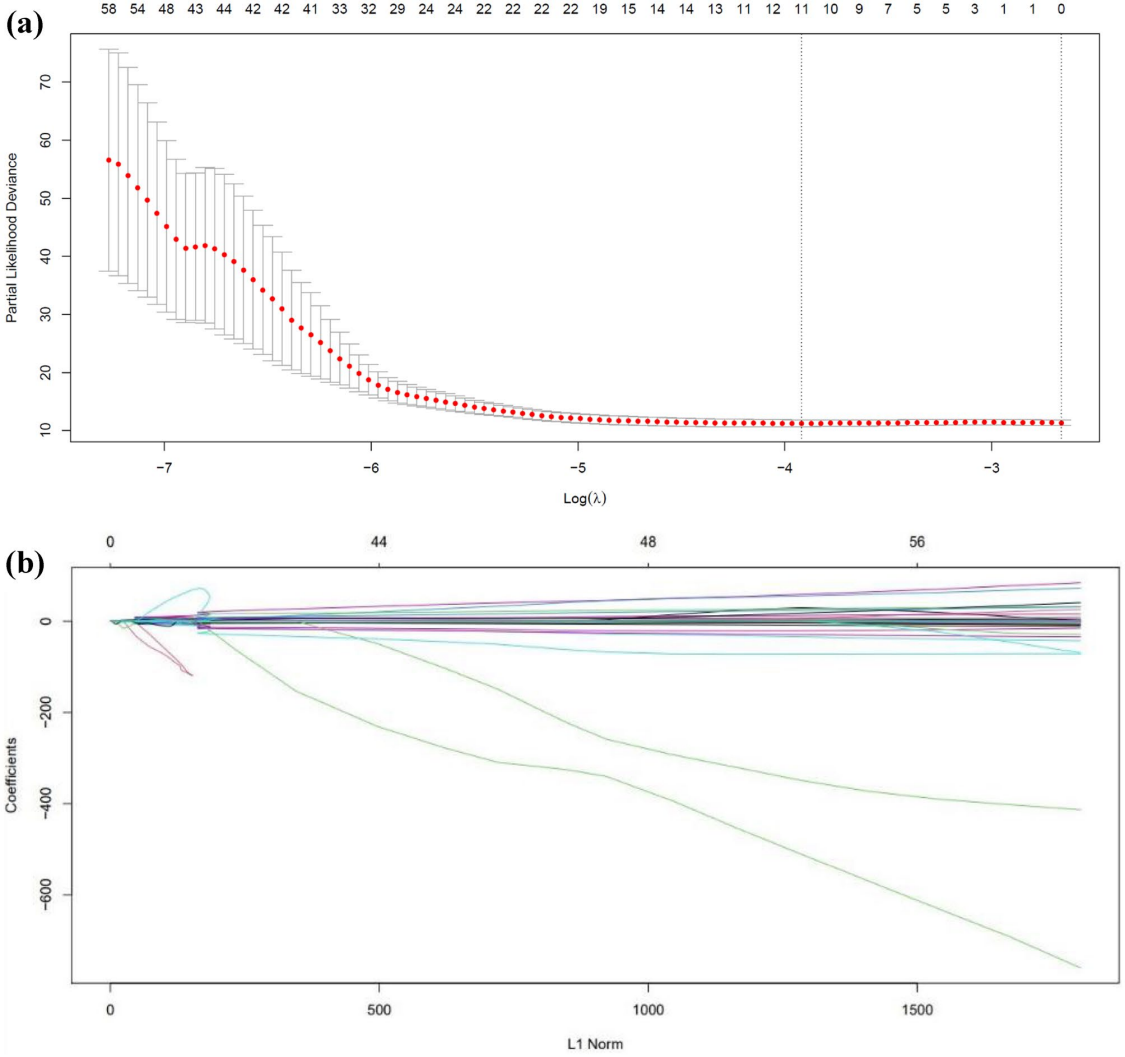
(**a**) Plot of the ten-fold cross-validation for identification of the optimal lambda (tuning parameter) based on minimizing the partial likelihood deviance error for our image features. (**b**) Plot of non-zero coefficients or the selected image features against the L1 norm penalty

Based on the results of the elastic net penalized Cox regression with ten-fold cross-validation and the fixed value of the hyper-parameter (*α* = 0.5), the identified optimal value of the tuning parameter ($\lambda$) was 0.01986259. For example, Fig. 2a presents the plot of our ten-fold cross-validation for the identification of optimal Log (*λ*) based on the minimization of partial likelihood deviance error for image features. The plot of features with non-zero coefficients (e.g. selected features) against the L1 norm is provided in Fig. 2b.

**Fig. 3:**
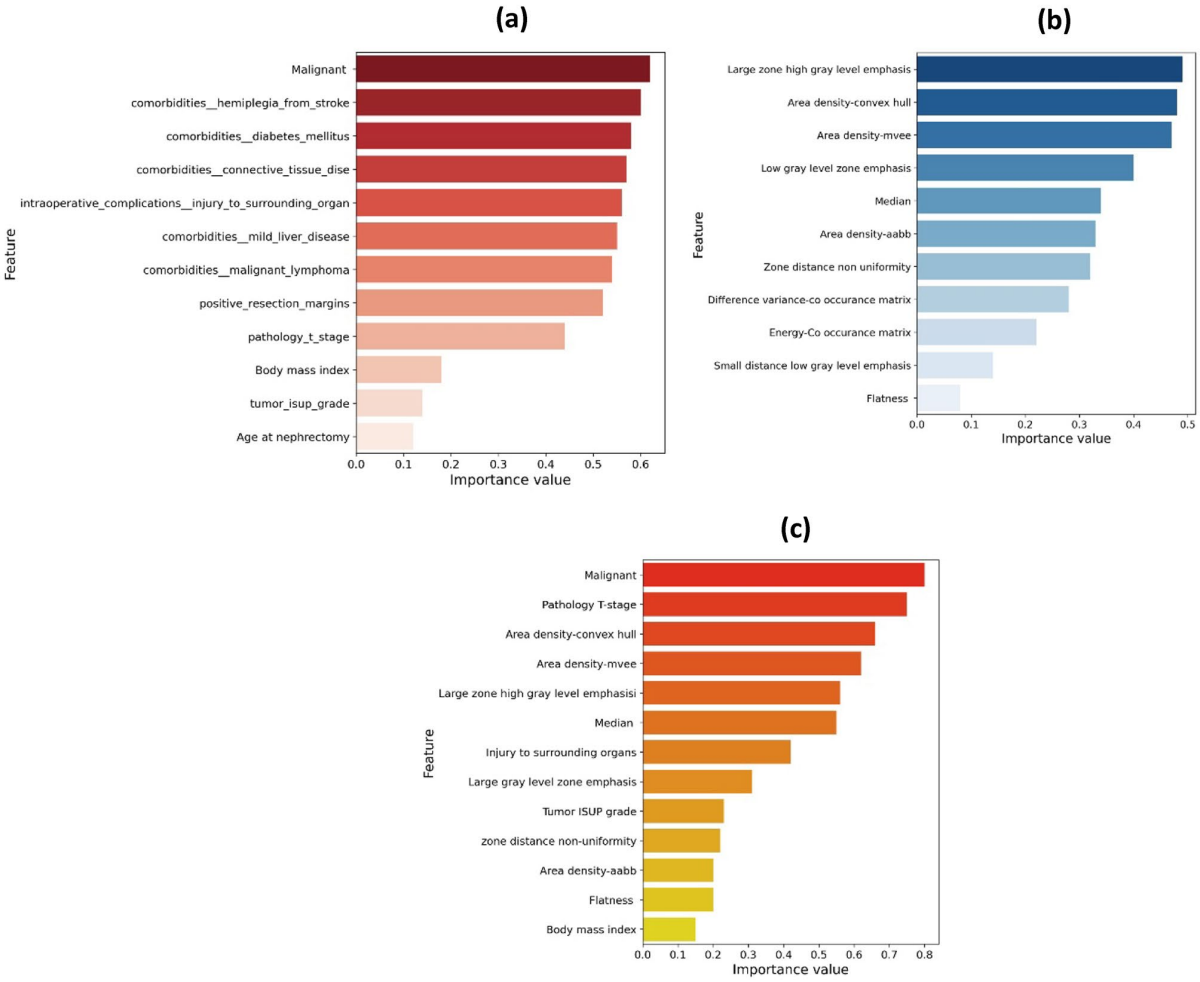
Selected radiomic and clinical features (non-zero coefficients) by elastic-net penalized Cox regression, “glmnet” R package (hyperparameter alpha = 0.5), with the tenfold cross-validation method (**a**) Clinical features. (**b**) Radiomic features. (**c**) Radiomic + clinical features

After fitting the model for radiomic, clinical, and clinical + radiomic data, features with non-zero coefficients were listed according to their importance value. The results are presented in Fig. 3. From 225 radiomic features, 59 clinical, and 284 combined features, 11, 12, and 13 features were respectively selected based on their non-zero coefficients and were ranked by their importance value. According to Fig. 3, malignancy had the highest importance value (62%) among the selected clinical features. Besides, almost half of the selected clinical features are associated with comorbidities. Among the selected radiomic features, large zone high gray level emphasis, area density (convex hull), and area density (MVEE) had the highest importance values (49%, 48%, and 47%, respectively). For the combination of radiomic and clinical features, malignancy, phatology T-stage, and area density-convex hull had the highest importance values (80%, 75%, and 66%, respectively).

### Model Selection and Survival Analysis

Table 1 summarizes the results of fitting the AFT shared frailty models to the radiomic, clinical, and radiomic + clinical features. According to this table, the model with Gamma frailty distribution and Weibull baseline distribution has the optimal performance for the radiomic signatures. The corresponding AIC and BIC values are 171 and 215, respectively (identified in bold). The same distributions also apply for the clinical and radiomic + clinical signatures corresponding to the lowest (AIC and BIC) values of (183, 230), and (160, 203), respectively.

**Table 1:** Comparison of AFT shared-frailty models by AIC and BIC (Bold cell for AIC and BIC indicated the best AFT shared-frailty model for radiomic and clinical data)

Model	Baseline distribution	Frailty distribution	AIC	BIC
Radiomics	Exponential	Gamma	172	215
		Inverse Gaussian	173	216
	Weibull	Gamma	**171**	**215**
		Inverse Gaussian	173	220
	Lognormal	Gamma	176	219
		Inverse Gaussian	174	221
	Log-logistic	Gamma	172	216
		Inverse Gaussian	173	218
Clinical	Exponential	Gamma	184	233
		Inverse Gaussian	183	231
	Weibull	Gamma	**183**	**230**
		Inverse Gaussian	184	232
	Lognormal	Gamma	189	239
		Inverse Gaussian	190	240
	Log-logistic	Gamma	184	234
		Inverse Gaussian	185	234
Radiomic + Clinical	Exponential	Gamma	162	208
		Inverse Gaussian	164	210
	Weibull	Gamma	**160**	**203**
		Inverse Gaussian	167	211
	Lognormal	Gamma	166	210
		Inverse Gaussian	164	212
	Log-logistic	Gamma	164	209
		Inverse Gaussian	168	213

The results of AFT Weibull shared-frailty model with bootstrapping are presented in Table 2. According to these results, tumor ISUP (International Society of Urologic Pathologists) grade, tumor malignancy, body mass index, and pathology t-stage were the most significant predictors of OS among the clinical features (*p* < 0.002, < 0.02, < 0.05, and < 0.02, respectively). The most significant predictors of OS between the selected radiomic features were flatness, area density (MVEE), and median (*p* < 0.02, < 0.02, and < 0.05, respectively). For radiomic + clinical model, a combination of all aforementioned features is a significant predictor of overall survival. According to Table 2 for multivariable models for the selected features, the model based on selected radiomic features has better prognostic power in comparison with the clinical model. The AIC and BIC for radiomic model were 171 and 215, whereas they were 183 and 230 for the clinical model, respectively. A combination of clinical and radiomic features resulted in a better prognostic model with AIC and BIC of 160 and 203, respectively, which is significantly better than the clinical model (Table 2).

**Table 2:** The accelerated failure time (AFT) Weibull shared-frailty (Gamma distribution) multivariable model for the selected features by the “survival” and “frailtypack” R packages with 1000 bootstrap samples

Feature	Selected variables	Feature type	Adj. *p*-value	Coefficient (SE)	Log likelihood (*p*-value)	AIC, BIC
Radiomics	Flatness	Morphological	0.013	9.28 (3.72)	−72.87 (0.009)	171,215
	Area density (AABB)	Morphological	0.139	−10.77 (7.25)		
	Area density (MVEE)	Morphological	0.019	−6.51 (2.76)		
	Area density (convex hull)	Morphological	0.587	−3.09 (5.69)		
	Median	Statistical	0.032	0.026 (0.012)		
	Difference variance (co-occurance matrix, 3D, averaged)	Texture	0.113	0.098 (0.062)		
	Energy (co-occurance matrix, 3D, averaged)	Texture	0.239	6.55 (5.56)		
	Low gray level zone emphasis (size zone matrix, 3D)	Texture	0.253	2.12 (2.35)		
	Large zone high gray level emphasis (size zone matrix, 3D)	Texture	0.823	−2.1 (9.41)		
	Small distance low gray level emphasis (distance zone matrix, 3D)	Texture	0.694	−1.99 (2.64)		
	Zone distance non uniformity (distance zone matrix, 3D)	Texture	0.650	0.0003 (0.0006)		
	Alpha	-	-	0.784 (0.146)		
	Frailty	-	-	0.03 (0.006)		
Clinical	Body mass index	Clinical	0.034	−0.081 (0.038)	−80.844 (0.003)	183,230
	Age at nephrectomy	Clinical	0.135	0.026 (0.017)		
	comorbidities__connective_tissue_dise (yes vs. no)	Clinical	0.803	−3.01 (12.07)		
	comorbidities__diabetes_mellitus (yes vs. no)	Clinical	0.825	−0.138 (0.624)		
	comorbidities__hemiplegia_from_stroke (yes vs. no)	Clinical	0.745	−3.0 (12.01)		
	comorbidities__malignant_lymphoma (yes vs. no)	Clinical	0.724	−3.02 (8.55)		
	comorbidities__mild_liver_diseas (yes vs. no)	Clinical	0.596	−3.08 (5.81)		
	intraoperative_complications__injury_to_surrounding_organ (yes vs. no)	Clinical	0.703	−3.02 (7.93)		
	Malignant (yes vs. no)	Clinical	0.017	3.14 (1.48)		
	pathology_t_stage (4 vs. 1)	Clinical	0.018	8.87 (4.22)		
	tumor_isup_grade 4 vs. 1)	Clinical	0.001	3.58 (1.05)		
	positive_resection_margins (yes vs. no)	Clinical	0.327	1.55 (3.46)		
	Alpha	-	-	0.769 (0.144)		
	Frailty	-	-	0.143 (0.071)		
Radiomic + Clinical	malignant		0.017	3.14 (1.48)	−70.01 (0.006)	160,203
	pathology_t_stage	Clinical	0.018	8.87 (4.22)		
	Body mass index	Clinical	0.014	−0.083 (0.038)		
	positive_resection_margins	Clinical	0.359	1.14 (3.16)		
	tumor_isup_grade	Clinical	0.002	3.19 (1.08)		
	Area density (convex hull)	Morphological	0.285	−3.18 (5.60)		
	Area density (MVEE)	Morphological	0.019	−6.51 (2.76)		
	Area density (AABB)	Morphological	0.073	−10.53 (7.25)		
	Large zone high gray level emphasis (size zone matrix, 3D)	Texture	0.823	−2.1 (9.41)		
	Low gray level zone emphasis (size zone matrix, 3D)	Texture	0.181	2.14 (2.35)		
	Zone distance non uniformity (distance zone matrix, 3D)	Texture	0.650	0.0003 (0.0006)		
	Flatness	Morphological	0.021	7.56 (3.72)		
	intraoperative_complications__injury_to_surrounding_organ	Clinical	0.703	−3.02 (7.93)		
	Median	Statistical	0.004	0.032 (0.012)		
	Alpha	-	-	0.788 (0.150)		
	Frailty	-	-	0.103 (0.056)		

Alpha is the shape parameter in the Weibull model. Frailty (sigma) is the standard deviation of gamma distribution in the Weibull AFT shared-frailty gamma model

*Adj. p-value p*-value adjusted by Benjamini and Hochberg method, *SE* standard error, *AIC* Akaike information criteria, *BIC* Bayesian information criteria

**Fig. 4:**
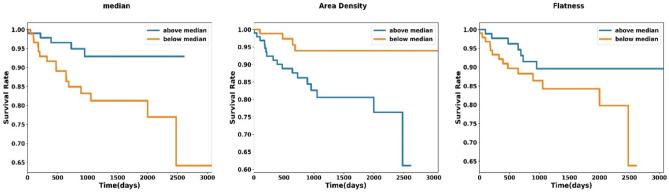
Kaplan Meier plot of the significant radiomic features

**Fig. 5:**
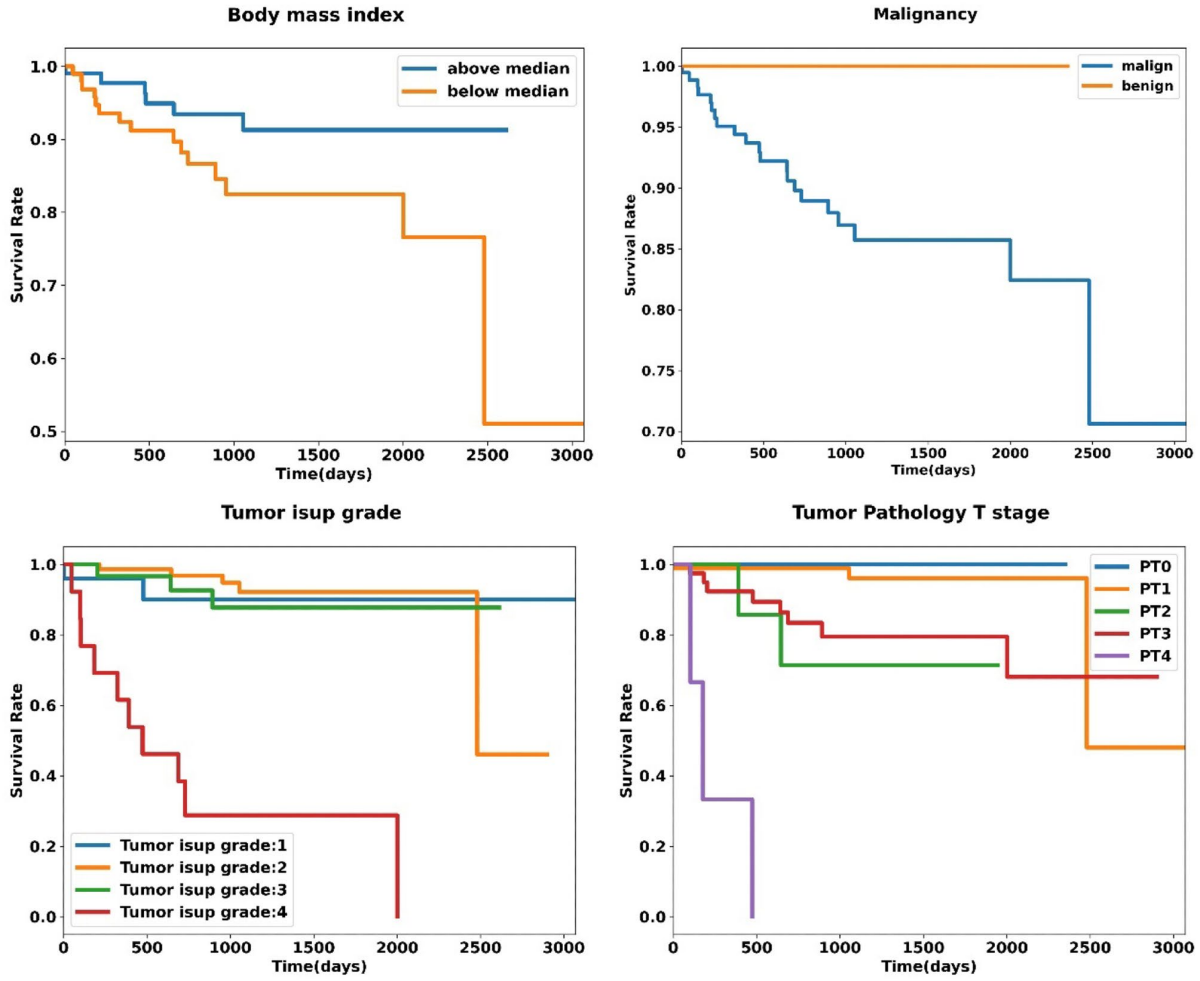
Kaplan Meier plots of the significant clinical features

The Kaplan Meier plot of significant radiomic and clinical features for OS are shown in Figs. 4 and 5, respectively. According to Fig. 4, lower values of median (median 77) and flatness (median 0.74) and higher values of area density (median 1.03) are correlated with poor OS. Figure 5 indicates that malignant lesions or lesions with higher isup grade, lower body mass index, and higher pathology T-stage are significantly associated with poor survival rate.

## Discussion

Radiomic analysis has emerged as a promising tool in the diagnosis, management, and survival time prediction of different types of cancer. A number of studies focused on the use of this tool for prognostication and survival prediction. Recently, Bologna et al. reported that MRI-based radiomics in patients with nasopharyngeal cancer can improve the prognostic capability when added to the clinical features [42]. Another study also concluded that there is the possibility to use extracted radiomic features of locally advanced non-small cell lung cancers to predict PFS [43]. Other studies have confirmed the use of radiomic features for determining OS in a variety of cancers, including pancreatic and hepatic cancers [44, 45]. In this work, we built prognostic models based on clinical, radiomic, and clinical + radiomic features for survival analysis of surgically treated RCC patients. Three radiomic features belonging to morphological and first-order statistical features and four clinical features were significant predictors of overall survival. Our findings suggest that the model based on radiomic features alone has better prognostic power compared with the clinical model and adding radiomic features to clinical features resulted in the best performance.

A limited number of studies investigated the association between CT texture features and OS or PFS in RCC patients [46]. Goh et al. [47] investigated the predictive power of CT texture features in the assessment of response to targeted therapy in metastatic renal cell carcinoma. Based on their results, both entropy and uniformity before the treatment and the percentage of change in uniformity were associated with time-to-progression. However, their study used a small sample size consisting of 39 patients. Lubner et al. [48] extracted CT texture features from images of 157 untreated RCC patients. They used Cox proportional hazard regression for time-to-event response. According to their results, an increase in the mean of positive pixels is associated with a shorter survival time. They also included different subtypes of RCCs but the type of treatment was not specified. Haider et al. [9] conducted a CT texture analysis for the prediction of OS and PFS in patients with clear cell RCC treated with Sunitinib. Their results demonstrated a significant association between entropy/size-normalized standard deviation and OS. However, the study population was small and included only 40 patients. In a study conducted by Nazari et al. [49], performance of radiomic models through renal cell carcinoma prognostication was investigated. However, patients were divided into high and low- risk groups (data was dichotomized) based on 5-year follow-up rather than a continuous time-to-event survival analysis. The best classifier based on a combination of clinical and radiomic features achieved an area under the receiver operating curve and accuracy with 95% confidence interval of 0.95–0.98 and 0.93–0.98, respectively. In a recent study, Li et al. [50] investigated the confounding factors of radiomic signature for predicting survival outcome in patients with clear cell RCC. They reported that a radiomic signature model independent of tumor size and CT slice thickness-related features is more reliable for survival analysis in RCC patients.

To the best of our knowledge, our study is the first one exploring the contribution of radiomic features in the prognosis of overall survival in RCC patients treated with partial or radical nephrectomy. Elastic-net penalized Cox regression was applied for dimensional reduction of the features by removing irrelevant features to handle the minimal-optimal problem. Owing to the lack of a homogeneous population and since all patients are not subject to the same risk, the results of the Cox proportional hazard model are unreliable and biased. Hence, the AFT model is a suitable alternative to the proportional hazard model to assess the effect of the selected radiomic and clinical features on OS [51]. Moreover, a frailty model is a robust tool to introduce random effects shared by subjects in the same group to the model, in correlated or clustered survival time data. It also induces dependence among the correlated or clustered failure time data. Conversely, the bootstrapping resampling method is a good strategy for robust inference of AFT shared-frailty regression coefficients. Therefore, we used AFT shared-frailty model with 1000 bootstrapping samples to assess the effects of selected features on overall survival time. In this work, we demonstrated that imaging biomarkers, including tumor flatness and area density which belong to morphological features and median from the statistical category, are predictive of overall survival after partial or radical nephrectomy. In addition, among clinical features, tumor ISUP grade, malignancy, pathology t-stage, and body mass index are statistically significant predictors of OS. Among all significant image biomarkers and clinical features, tumor ISUP grade is more correlated with OS (*p* < 0.002). One of the highlights of our study with respect to previous studies investigating the correlation between imaging biomarkers and OS in RCC patients is that our study population was significantly larger. Furthermore, previous studies only considered a limited number of CT texture features, whereas our study was more comprehensive as we explored the potential predictive power of 225 radiomic features.

Our findings seem to indicate that three radiomic features belonging to morphological and statistical features category, including flatness, median, and area density, were significant predictors of OS for patients with RCC. Flatness (the square root of the ratio of least and major axis lengths) refers to the flatness of a volume relative to its length. One and near 1 values indicate that the volume is non-flat or spherical [28]. Higher values of flatness, i.e., more sphericity, are associated with better survival outcome (Fig. 4). Several studies confirmed that sphericity and features derived from sphericity are significant prognostic factors for different cancer types. Davey et al. [52] investigated the correlation between sphericity and other clinical prognostic factors in non-small cell lung cancer patients. They reported that sphericity is strongly associated with overall survival and correlates with clinical factors, such as tumor volume, N stage, and T-stage. Low sphericity is associated with large tumor volume and consequently worse prognosis. Moreover, sphericity is lower for higher T and N stages compared to T1 and N0, and nodal involvement within GTV results in more complex shape and lower sphericity. This is in agreement with previous studies which reported significant correlation between sphericity and treatment outcome or overall survival for esophageal cancer [53], meningioma [54], breast cancer [55], and glioblastoma [56].

Median intensity is another significant radiomic feature highlighted in our results. As can be seen in Fig. 4, a higher median intensity is associated with better prognosis. Higher intensities in contrast-enhanced CT images can be related to angiogenesis. In the context of renal cell carcinoma, the relation between angiogenesis and overall survival is controversial. Several studies investigated the correlation between microvascular density, which is a surrogate of histomorphologic marker of tumor angiogenesis, and overall survival in RCC patients. Some of these reports have shown that higher tumor vascularity has no correlation with RCC patients’ overall survival [57, 58]. Other studies reported that higher microvascular density is associated with poor survival [59, 60]. In a more recent study by Zhu et al. [61], the correlation between tumor enhancement and Fuhrman grade was investigated on contrast enhanced CT images of 255 patients who underwent partial or radical nephrectomy. Tumor enhancement on corticomedullary phase is correlated with microvascular density [62]. The results of this study showed that lower tumor enhancement is associated with higher tumor grade. This is in line with our results regarding lower median intensity, which is correlated with poor survival. One reasonable explanation for inverse association between median intensity and survival is the presence of histologic necrosis within the tumor. Tumor histologic necrosis is hypovascular regions containing dead cells and is correlated with tumor aggressiveness, poor prognosis, higher tumor size, stage, and grade [63, 64].

Another significant morphological feature is area density-mvee. Area density is the fraction of ROI surface area and the surface area of the smallest ellipsoid enclosing the ROI [28]. Higher area density is associated with worse overall survival (Fig. 4). Area density can be correlated with tumor size and a number of studies confirmed that tumor size is a strong and independent predictor of overall survival in patients with renal cell carcinoma [63, 65, 66].

Among clinical features, body mass index (BMI) is one of the significant predictors of overall survival. Higher BMI is correlated with better prognosis (Fig. 5). Although obesity is one of the risk factors of RCC, a higher BMI is associated with better postoperative survival in patients who underwent radical or partial nephrectomy [67]. One possible explanation is that overweight patients can better deal with post-surgery stress due to better metabolic state, energy, and nutritional reserves. Also, a higher level of adiponectin in RCC patients with lower BMI is associated with poor survival and aggressive cell proliferation [68].

Pathological T-stage is another significant clinical feature and a higher pathology T-stage is correlated with poor prognosis (Fig. 5). In fact, higher T-stages relate to larger tumor or more amount of spread to nearby tissues, which results in worse survival outcome. This is in line with previous studies which investigated the correlation between tumor grade and overall survival in RCC patients [69].

Tumor ISUP grade is also a significant predictor of overall survival in patients with RCC. ISUP grade 4 has the worse prognosis in comparison to ISUP grades 1 to 3 (Fig. 5). However, overall survival for ISUP grades 1 to 3 is somewhat similar. In a study by Li-Yan et al. [70] on 842 RCC patients, it has been shown that there is a significant association between tumor ISUP grade 4 and decreased overall survival but there is no significant difference in outcome for tumors of ISUP grades 1 to 3.

The main limitation of the current study was the retrospective nature of the study and data heterogeneity since image acquisitions were performed at referring institutions with different scanners and acquisition protocols. Some studies have reported that different scanners, image acquisition protocols, and reconstruction techniques may lead to radiomic features variability [71, 72].

## Conclusions

Along with important clinical features, e.g., tumor heterogeneity and the ISUP grade, which are routinely used as a predictor of OS, we demonstrated that imaging biomarkers, such as tumor flatness, area density, and median, are significantly correlated with OS of RCC patients treated by partial or radical nephrectomy.

## Supplementary Information

Below is the link to the electronic supplementary material.

Supplementary file1 (PDF 538 KB)

## Data Availability

The dataset used in this work are available from The Cancer Imaging Archive (TCIA) https://www.cancerimagingarchive.net/.

## Code Availability

The Standardized Environment for Radiomics Analysis (SERA) package used in this work is available on https://qurit.ca/software/sera/.
