# Supplementary material for: Overall Survival Prediction in Renal Cell Carcinoma Patients Using Computed Tomography Radiomic and Clinical Information
Source: J Digit Imaging. 2021 Aug 11;34(5):1086–98. doi: 10.1007/s10278-021-00500-y (PMC8554934; doi:10.1007/s10278-021-00500-y)
Supplement: Supplementary file 1 — Supplementary file1 (PDF 538 KB) [file 10278_2021_500_MOESM1_ESM.pdf]

Table 1. Summary of clinical features.

Characteristic	Number		Characteristic	Number	
Sex			Comorbidity/leukemia		
	Male	123 (58%)		Negative	209 (99.5%)
	Female	87 (41%)		Positive	1 (0.5%)
Age at nephrectomy	58.3 ± 14.3		Comorbidity/hemiplegia from stroke		
Malignancy				Negative	209 (99.5%)
	Malignant	192 (91%)		Positive	1 (0.5%)
	Non-malignant	18 (9%)	Comorbidity/ chronic kidney disease		
ISUP grade				Negative	194 (92.3%)
	1	26 (12%)		Positive	16 (7.6%)
	2	93 (44%)	Comorbidity/ mild liver disease		
	3	39 (18%)		Negative	206 (98%)
	4	14 (6%)		Positive	4 (2%)
	Not answered	38 (18%)	Comorbidity/metastatic solid tumor		
Surgical procedure				Negative	198 (94%)
	Partial nephrectomy	140 (66%)		Positive	12 (6%)
	Radical nephrectomy	70 (33%)	Comorbidity/localized solid tumor		
Vital status				Negative	181 (86%)
	Censored	189 (90%)		Positive	29 (14%)
	Dead	21 (10%)	Smoking history		
Comorbidity/ congestive heart failure				Current smoker	34 (16%)
	Negative	204 (97%)		Never smoked	100 (48%)
	Positive	6 (2.8%)		Previous smoker	76 (36%)
Comorbidity/myocardial infraction			Comorbidity/aids		
	Negative	201 (95%)		Negative	210 (100%)
	Positive	9 (4.2%)	Comorbidity/moderate to severe liver disease		
Comorbidity/connective tissue disease				Negative	209 (99.5%)
	Negative	209 (99.5%)		Positive	1 (0.5%)
	Positive	1 (0.4%)			

Comorbidity/copd			Alcohol use	More then two daily	13 (6.1%)
	Negative	199 (94.7%)		Never or not in last 3 month	95 (45.2%)
	Positive	11 (5.3%)		Two or less daily	102 (48.5%)
Comorbidity/dementia			Chewing tobacco use		
	Negative	210 (100%)		Never or not in last 3 month	209 (99.5%)
Comorbidity/cerebrovascular disease				Quit in last 3 month	1 (0.5%)
	Negative	206 (98%)	Interoperative complication/cardiac event		
	Positive	4 (2%)		Negative	210 (100%)
Comorbidity/peripheral vascular disease			Interoperative complication/injury to surrounding organs		
	Negative	201 (95.7%)		Negative	207 (98.5%)
	Positive	9 (4.2%)		Positive	3 (1.5%)
Comorbidity/ diabetes mellitus with end organ damage			Intraoperative complication/blood transfusion		
	Negative	208 (99%)		Negative	207 (98.5%)
	Positive	2 (1%)		Positive	3 (1.5%)
Comorbidity/uncomplicated diabetes mellitus			Pathology m-stage		
	Negative	172 (81.9%)		0	93 (44.2%)
	Positive	38 (18%)		1	17 (8%)
Comorbidity/ peptic ulcer disease				X	100 (47.6%)
	Negative	209 (99.5%)	Pathology n-stage		
	Positive	1 (0.5%)		0	90 (42.8%)
Comorbidity/ malignant lymphoma				1	6 (2.8%)
	Negative	208 (99%)		X	114 (54.2%)
	Positive	2 (1%)	Pathology t-stage		
Tumor necrosis				1a	92 (43.8%)
	Negative	144 (68.5%)		1b	41 (19.5%)
	Positive	43 (20.4%)		2a	9 (4.2%)
	NA	23 (10.9%)		2b	3 (1.4%)
	Angiomyolipoma	5 (2.3%)		0	149 (70.9%)
	Chromophobe	19 (9%)		1	27 (12.8%)

Histologic subtype	Clear-cell-papillary -rcc	4 (2%)	Clavien surgical complications	2	15 (7.1%)
	Clear cell rcc	143 (68%)		4	4 (1.9%)
	Mest	2 (1%)		5	1 (0.5%)
	Multilocular cystic rcc	1 (0.5%)		3a	6 (2.8%)
	Oncocytoma	10 (4.7%)		3b	7 (3.3%)
	Papillary	21 (10%)		NA	1 (0.5%)
	Rcc unclassified	2 (1%)	er- visit		
	Spindle cell neoplasm	1 (0.5%)		Negative	191(90.9%)
	Urothelial	1 (0.5%)		Positive	17 (8%)
	Wilms	1 (0.5%)		NA	2 (1%)
Cytoreductive			Positive resection margins		
	Negative	193 (91.9%)		Negative	199 (94.7%)
	Positive	17 (8%)		Positive	11 (5.2%)
Surgery type			Readmission		
	Laparoscopic	23 (13.3%)		Negative	187 (89%)
	Open	60 (28.5%)		Positive	21 (10%)
	Robotic	122 (58%)		NA	2 (1%)
Surgical approach			Hospitalization	3.6 ± 2.99	
	Retroperitoneal	39 (18.5%)	Radiographic size	4.77 ± 3.1	
	Transperitoneal	171 (81.5%)	Pathologic size	4.71 ±3.21	
Body mass index	31.17 ± 6.46		Estimated blood loss	313.96 ± 351.2	
Operative time	242.96 ± 99.33		Vital days after surgery	956.7 ± 778.79	
Last preop egfr	73.93 ± 14.87		Last preop egfr/ days before surgery	23.38 ± 22.11	
First postop egfr	64.1 ± 18.97		First postop egfr/days after surgery	437.59 ± 604.99	
Last postop egfr	64.88 ± 19.5		Last postop egfr/days after surgery	1047.96 ± 726.04	
Pack years	9.9 ± 24.26		Age when quit smoking	42.1 ± 13.37	
Ischemia time	19.96 ± 11.4				

Table 2. Radiomics features extracted from CT images.

Statistics	Intensity histogram	Co-occurrence matrix (3D, averaged)
Mean (stat_mean)	Mean (ih_mean)	Joint maximum (cm_joint_max_3D_avg)
Variance (stat_var)	Variance (ih_var)	Joint average (cm_joint_avg_3D_avg)
Skewness (stat_skew)	Skewness (ih_skew)	Joint variance (cm_joint_var_3D_avg)
(Excess) kurtosis (stat_kurt)	Kurtosis (ih_kurt)	Joint entropy (cm_joint_entr_3D_avg)
Median (stat_median)	Median (ih_median)	Difference average (cm_diff_avg_3D_avg)
Minimum (stat_min)	Minimum (ih_min)	Difference variance (cm_diff_var_3D_avg)
10th percentile (stat_p10)	10th percentile (ih_p10)	Difference entropy (cm_diff_entr_3D_avg)
90th percentile (stat_p90)	90th percentile (ih_p90)	Sum average (cm_sum_avg_3D_avg)
Maximum (stat_max)	Maximum (ih_max)	Sum variance (cm_sum_var_3D_avg)
Interquartile range (stat_iqr)	Mode (ih_mode)	Sum entropy (cm_sum_entr_3D_avg)
Range (stat_range)	Interquartile range (ih_iqr)	Angular second moment (cm_energy_3D_avg)
Mean absolute deviation (stat_mad)	Range (ih_range)	Contrast (cm_contrast_3D_avg)
Robust mean absolute deviation (stat_rmad)	Mean absolute deviation (ih_mad)	Dissimilarity (cm_dissimilarity_3D_avg)
Median absolute deviation (stat_medad)	Robust mean absolute deviation (ih_rmad)	Inverse difference (cm_inv_diff_3D_avg)
Coefficient of variation (stat_cov)	Median absolute deviation (ih_medad)	Inverse difference normalised (cm_inv_diff_norm_3D_avg)
Quartile coefficient of dispersion (stat_qcod)	Coefficient of variation (ih_cov)	Inverse difference moment (cm_inv_diff_mom_3D_avg)
Energy (stat_energy)	Quartile coefficient of dispersion (ih_qcod)	

Root mean square (stat_rms)	Entropy (ih_entropy)	Inverse difference moment normalised (cm_inv_diff_mom_norm_3D_avg)
	Uniformity (ih_uniformity)	Inverse variance (cm_inv_var_3D_avg)
**Morphology**	Maximum histogram gradient (ih_max_grad)	Correlation (cm_corr_3D_avg)
Volume (mesh-based) (morph_volume)	Maximum gradient grey level (ih_max_grad_g)	Autocorrelation (cm_auto_corr_3D_avg)
Volume (counting) (morph_vol_approx)	Minimum histogram gradient (ih_min_grad)	Cluster tendency (cm_clust_tend_3D_avg)
Surface area (morph_area_mesh)	Minimum gradient grey level (ih_min_grad_g)	Cluster shade (cm_clust_shade_3D_avg)
Surface to volume ratio (morph_av)		Cluster prominence (cm_clust_prom_3D_avg)
Compactness 1 (morph_comp_1)	**Intensity volume histogram**	Information correlation 1 (cm_info_corr1_3D_avg)
Compactness 2 (morph_comp_2)		Information correlation 2 (cm_info_corr2_3D_avg)
Spherical disproportion (morph_sph_dispr)	Volume fraction at 10% intensity (ivh_v10)	Joint maximum (cm_joint_max_3D_comb)
Sphericity (morph_sphericity)	Volume fraction at 90% intensity (ivh_v90)	Joint average (cm_joint_avg_3D_comb)
Asphericity (morph_asphericity)	Intensity at 10% volume (ivh_i10)	Joint variance (cm_joint_var_3D_comb)
Centre of mass shift (morph_com)	Intensity at 90% volume (ivh_i90)	Joint entropy (cm_joint_entr_3D_comb)
Maximum 3D diameter (morph_diam)	Volume fraction difference between 10% and 90% intensity (ivh_diff_v10_v90)	Difference average (cm_diff_avg_3D_comb)
Major axis length (morph_pca_maj_axis)		Difference variance (cm_diff_var_3D_comb)
Minor axis length (morph_pca_min_axis)	Intensity difference between 10% and 90% volume (ivh_diff_i10_i90)	Difference entropy (cm_diff_entr_3D_comb)
Least axis length (morph_pca_least_axis)	Area under the IVH curve (ivh_auc)	Sum average (cm_sum_avg_3D_comb)
Elongation (morph_pca_elongation)		Sum variance (cm_sum_var_3D_comb)
Flatness (morph_pca_flatness)	**Local intensity**	Sum entropy (cm_sum_entr_3D_comb)
Volume density (AABB) (morph_vol_dens_aabb)	Local intensity peak (loc_peak_loc)	Angular second moment (cm_energy_3D_comb)

Area density (AABB) (morph_area_dens_aabb)

Volume density (OMBB) (morph_vol_dens_ombb)

Area density (OMBB) (morph_area_dens_ombb)

Volume density (AEE) (morph_vol_dens_aee)

Area density (AEE) (morph_area_dens_aee)

Volume density (MVEE) (morph_vol_dens_mvee)

Area density (MVEE) (morph_area_dens_mvee)

Volume density (convex hull) (morph_vol_dens_conv_hull)

Area density (convex hull) (morph_area_dens_conv_hull)

Integrated intensity (morph_integ_int)

Moran's I index (morph_moran_i)

Geary's C measure (morph_geary_c)

Moment Invariant

J1 (mi_j1)

Q (mi_q)

J2 (mi_j2)

J3 (mi_j3)

B3 (mi_b3)

Global intensity peak (loc_peak_glob)

Size zone matrix (3D)

Small zone emphasis (szm_sze_3D)

Large zone emphasis (szm_lze_3D)

Low grey level emphasis (szm_lgze_3D)

High grey level emphasis (szm_hgze_3D)

Small zone low grey level emphasis (szm_szlge_3D)

Small zone high grey level emphasis (szm_szhge_3D)

Large zone low grey level emphasis (szm_lzlge_3D)

Large zone high grey level emphasis (szm_lzhge_3D)

Grey level non-uniformity (szm_glnu_3D)

Grey level non uniformity normalised (szm_glnu_norm_3D)

Zone size non-uniformity (szm_zsnu_3D)

Zone size non-uniformity normalised (szm_zsnu_norm_3D)

Zone percentage (szm_z_perc_3D)

Grey level variance (szm_gl_var_3D)

Zone size variance (szm_zs_var_3D)

Zone size entropy (szm_zs_entr_3D)

Contrast (cm_contrast_3D_comb)

Dissimilarity (cm_dissimilarity_3D_comb)

Inverse difference (cm_inv_diff_3D_comb)

Inverse difference normalised (cm_inv_diff_norm_3D_comb)

Inverse difference moment (cm_inv_diff_mom_3D_comb)

Inverse difference moment normalised (cm_inv_diff_mom_norm_3D_comb)

Inverse variance (cm_inv_var_3D_comb)

Correlation (cm_corr_3D_comb)

Autocorrelation (cm_auto_corr_3D_comb)

Cluster tendency (cm_clust_tend_3D_comb)

Cluster shade (cm_clust_shade_3D_comb)

Cluster prominence (cm_clust_prom_3D_comb)

Information correlation 1 (cm_info_corr1_3D_comb)

Information correlation 2 (cm_info_corr2_3D_comb)

Run length matrix (3D, averaged)

Short runs emphasis (rlm_sre_3D_avg)

oJ1 (mi_oj1)

oQ (mi_oq)

oJ2 (mi_oj2)

oJ3 (mi_oj3)

oB3 (mi_ob3)

Neighbouring grey level dependence matrix (3D)

Coarseness (ngt_coarseness_3D)

Contrast (ngt_contrast_3D)

Busyness (ngt_busyness_3D)

Complexity (ngt_complexity_3D)

Strength (ngt_strength_3D)

Low dependence emphasis (ngl_lde_3D)

High dependence emphasis (ngl_hde_3D)

Low grey level count emphasis (ngl_lgce_3D)

High grey level count emphasis (ngl_hgce_3D)

Low dependence low grey level emphasis (ngl_ldlge_3D)

Low dependence high grey level emphasis (ngl_ldhge_3D)

Distance zone matrix (3D)

Small distance emphasis (dzm_sde_3D)

Large distance emphasis (dzm_lde_3D)

Low grey level emphasis (dzm_lgze_3D)

High grey level emphasis (dzm_hgze_3D)

Small distance low grey level emphasis (dzm_sdlge_3D)

Small distance high grey level emphasis (dzm_sdhge_3D)

Large distance low grey level emphasis (dzm_ldlge_3D)

Large distance high grey level emphasis (dzm_ldhge_3D)

Grey level non-uniformity (dzm_glnu_3D)

Grey level non-uniformity normalised (dzm_glnu_norm_3D)

Zone distance non-uniformity (dzm_zdnu_3D)

Zone distance non-uniformity normalised (dzm_zdnu_norm_3D)

Zone percentage (dzm_z_perc_3D)

Grey level variance (dzm_gl_var_3D)

Long runs emphasis (rlm_lre_3D_avg)

Low grey level run emphasis (rlm_lgre_3D_avg)

High grey level run emphasis (rlm_hgre_3D_avg)

Short run low grey level emphasis (rlm_srlge_3D_avg)

Short run high grey level emphasis (rlm_srhge_3D_avg)

Long run low grey level emphasis (rlm_lrlge_3D_avg)

Long run high grey level emphasis (rlm_lrhge_3D_avg)

Grey level non-uniformity (rlm_glnu_3D_avg)

Grey level non-uniformity normalised (rlm_glnu_norm_3D_avg)

Run length non-uniformity (rlm_rlnu_3D_avg)

Run length non-uniformity normalised (rlm_rlnu_norm_3D_avg)

Run percentage (rlm_r_perc_3D_avg)

Grey level variance (rlm_gl_var_3D_avg)

Run length variance (rlm_rl_var_3D_avg)

Run entropy (rlm_rl_entr_3D_avg)

Short runs emphasis (rlm_sre_3D_comb)

High dependence low grey level emphasis (ngl_hdlge_3D)<br>High dependence high grey level emphasis (ngl_hdhge_3D)<br>Grey level non-uniformity (ngl_glnu_3D)<br>Grey level non-uniformity normalised (ngl_glnu_norm_3D)<br>Dependence count non-uniformity (ngl_dcnu_3D)<br>Dependence count non-uniformity normalised (ngl_dcnu_norm_3D)<br>Dependence count percentage (ngl_dc_perc_3D)<br>Grey level variance (ngl_gl_var_3D)<br>Dependence count variance (ngl_dc_var_3D)<br>Dependence count entropy (ngl_dc_entr_3D)<br>Dependence count energy (ngl_dc_energy_3D)	Zone distance variance (dzm_zd_var_3D)<br>Zone distance entropy (dzm_zd_entr_3D)	Long runs emphasis (rlm_lre_3D_comb)<br>Low grey level run emphasis (rlm_lgre_3D_comb)<br>High grey level run emphasis (rlm_hgre_3D_comb)<br>Short run low grey level emphasis (rlm_srlge_3D_comb)<br>Short run high grey level emphasis (rlm_srhge_3D_comb)<br>Long run low grey level emphasis (rlm_lrlge_3D_comb)<br>Long run high grey level emphasis (rlm_lrhge_3D_comb)<br>Grey level non-uniformity (rlm_glnu_3D_comb)<br>Grey level non-uniformity normalised (rlm_glnu_norm_3D_comb)<br>Run length non-uniformity (rlm_rlnu_3D_comb)<br>Run length non-uniformity normalised (rlm_rlnu_norm_3D_comb)<br>Run percentage (rlm_r_perc_3D_comb)<br>Grey level variance (rlm_gl_var_3D_comb)<br>Run length variance (rlm_rl_var_3D_comb)<br>Run entropy (rlm_rl_entr_3D_comb)
